# Bone Marrow Recovery by Morphometry during Induction Chemotherapy for Acute Lymphoblastic Leukemia in Children

**DOI:** 10.1371/journal.pone.0126233

**Published:** 2015-05-11

**Authors:** Tuong-Vi Nguyen, Anna Melville, Shriram Nath, Colin Story, Stuart Howell, Rosemary Sutton, Andrew Zannettino, Tamas Revesz

**Affiliations:** 1 Erasmus University, Rotterdam, The Netherlands; 2 SA Pathology at Women’s and Children’s Hospital, Adelaide, Australia; 3 Women’s and Children’s Research Institute, Adelaide, Australia; 4 SA Pathology, Frome Road, Adelaide, Australia; 5 Data Management & Analysis Centre, Discipline of Public Health, University of Adelaide, Adelaide, Australia; 6 Children’s Cancer Institute Australia, Lowy Cancer Research Centre, UNSW, Randwick, NSW, Australia; 7 Faculty of Health Science, University of Adelaide, Adelaide, Australia; 8 Centre for Cancer Biology, SA Pathology, Adelaide, Australia; University of Heidelberg, GERMANY

## Abstract

Bone marrow architecture is grossly distorted at the diagnosis of ALL and details of the morphological changes that accompany response to Induction chemotherapy have not been reported before. While marrow aspirates are widely used to assess initial response to ALL therapy and provide some indications, we have enumerated marrow components using morphometric analysis of trephine samples with the aim of achieving a greater understanding of changes in bone marrow niches. Morphometric analyses were carried out in the bone marrow trephine samples of 44 children with ALL, using a NanoZoomer HT digital scanner. Diagnostic samples were compared to those of 32 control patients with solid tumors but without marrow involvement. Samples from patients with ALL had significantly increased fibrosis and the area occupied by bony trabeculae was lower than in controls. Cellularity was higher in ALL samples due to leukemic infiltration while the percentage of normal elements such as megakaryocytes, adipocytes, osteoblasts and osteoclasts were all significantly lower. During the course of Induction therapy, there was a decrease in the cellularity of ALL samples at day 15 of therapy with a further decrease at the end of Induction and an increase in the area occupied by adipocytes and the width of sinusoids. Reticulin fibrosis decreased throughout Induction. Megakaryocytes increased, osteoblasts and osteoclasts remained unchanged. No correlation was found between clinical presentation, early response to treatment and morphological changes. Our results provide a morphological background to further studies of bone marrow stroma in ALL.

## Introduction

Bone marrow aspiration and trephine biopsy are standard procedures in diagnosing acute lymphoblastic leukemia (ALL) in children. Bone marrow aspirations are also used at later time points during treatment to establish response to therapy including testing for minimal residual disease (MRD). While bone marrow aspirates are informative for most aspects of diagnostic value for leukemia patients, bone marrow trephine biopsy specimens have the advantage of showing the extent of disruption in bone marrow integrity associated with the development of leukemia and can be a valuable source for diagnostic tests in case of a ‘dry tap’.

At diagnosis, most children with ALL show extensive infiltration of the bone marrow spaces by leukemic blasts and there is very little evidence of normal haemopoiesis. With modern chemotherapy a rapid decrease of the leukemic blasts is seen by day 15 and usually the day 30 bone marrow aspirate shows remission with less than 5% blasts. Presentation marrows are also characterized by the presence of increased reticulin fibrosis. Noren-Nystrom et al reported that elevated reticulin fibrosis was a common finding in diagnosis trephine biopsy specimens which then returns to normal levels following response to chemotherapy [1]. We were able to confirm these findings [2] and also hypothesized that high reticulin fibre content in the marrow ‘anchors’ leukemic cells and is therefore associated with lower blood blast counts [3].

There is very little reported on the details of bone marrow morphological changes that accompany response to induction chemotherapy in ALL. As a first phase of studies examining the role of the stromal components of the marrow in ALL, we systemically reviewed trephine biopsies obtained from newly diagnosed patients with ALL at diagnosis, day 15 and at the end of induction and at diagnosis from control patients diagnosed with other cancers but no marrow involvement.

## Materials and Methods

### Ethics Statement

Our study complied with the guidelines of the Declaration of Helsinki. As such, the Human Research Ethics Committee of the Women’s and Children’s Hospital (HREC number 2250/3/13) approved the study. Parents for all patients provided written informed consent to examination of their child’s bone marrow trephine samples obtained during the process of diagnostic studies and to the recording of their de-identified follow-up data in the research data registry.

### Patients

Forty-four patients 1–17 years old at diagnosis with ALL and 32 controls of similar ages were included in the study between January 2009 and February 2012. Only patients who had good quality trephine biopsy specimens at diagnosis (with less than ~25% of the specimen affected by crash artifacts) were included in the analyses. The control patients were diagnosed with: Non-Hodgkin Lymphoma (n = 6), Hodgkin lymphoma (8), Neuroblastoma (5), Soft tissue tumour (7) and Ewing sarcoma (6).

The following protocols were used for the treatment of the leukemia patients: COG AALL0331, AALL0232, AALL0434 and the BFM-based Australian and New Zealand Children’s Haematogy and Oncology Group Study 8 (ANZCHOG ALL8). The induction part of these protocols was very similar. Clinical data were obtained from case notes and hospital electronic records.

BM trephine biopsies were obtained at diagnosis, day 15 and end of induction treatment (day 29 for COG studies and day 33 for Study 8) in 44 patients with ALL (B-cell precursor n = 39; T cell n = 5). Control trephine biopsy samples were only obtained at diagnosis and were only included in the study if the marrow contained no tumor cells.

Minimal Residual Disease status (MRD) was evaluated at the end of induction (day 29 or 33 depending on protocol) using either flow-cytometry [4] or by PCR [5,6]. Patients who had MRD levels >1x10-3 (>0.1%) at end of induction in all protocols were generally regarded as having high-risk for relapse but treatment recommendations were dependent on the immunophenotype and specific therapy protocol used. In general, high-risk patients were stratified into treatment with intensive high risk blocks and recommended stem cell transplantation.

### Trephine biopsy morphometry

**Fig 1:**
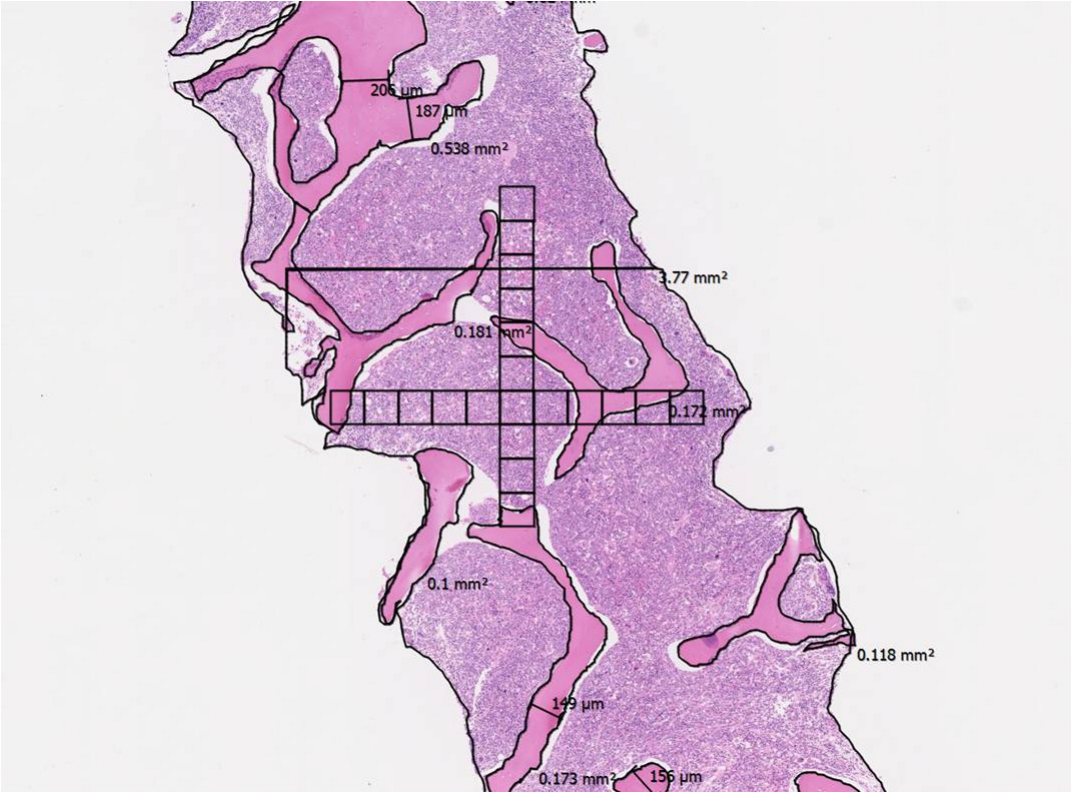
Example of surface measurements on a NanoZoomer specimen from a patient with newly diagnosed ALL. Magnification x1.25. The place and orientation of the 20 fields that were used for some of the morphometry measurements is also shown.

4-μm sections of the trephine biopsies were stained with hematoxylin & eosin (H&E) or by Gordon and Sweets’ method for reticulin fibres and scanned using a Hamamatsu NanoZoomer HT (Hamamatsu Photonics KK) digital scanner. The images were analyzed using a 10 x 10 square grid with 121 crossing points at a magnification of x40 (corresponding to a 0.02 mm^2^ area). 20 representative fields in the H&E preparations and 10 fields in the reticulin slides were selected at low magnification and analyzed at x40 (Fig 1). Fields were selected to give as close an approximation of the whole biopsy specimen as possible. In practice, this meant that a field was chosen randomly at low power and subsequently 10 fields were counted ‘in line’ with the first one, then 10 fields across, thus avoiding bias in the selection of fields. If this approach was not possible for a given specimen, fields were selected at low power to fit the confines of the biopsy specimen, then analyzed as above. Reticulin fibers were counted when they overlaid a crossing point, as described in Noren-Nystrom et al [1]. In our earlier report we compared reticulin fiber quantitation using the above method with that of the Bauermeister scale and found a very good correlation [2].

**Fig 2:**
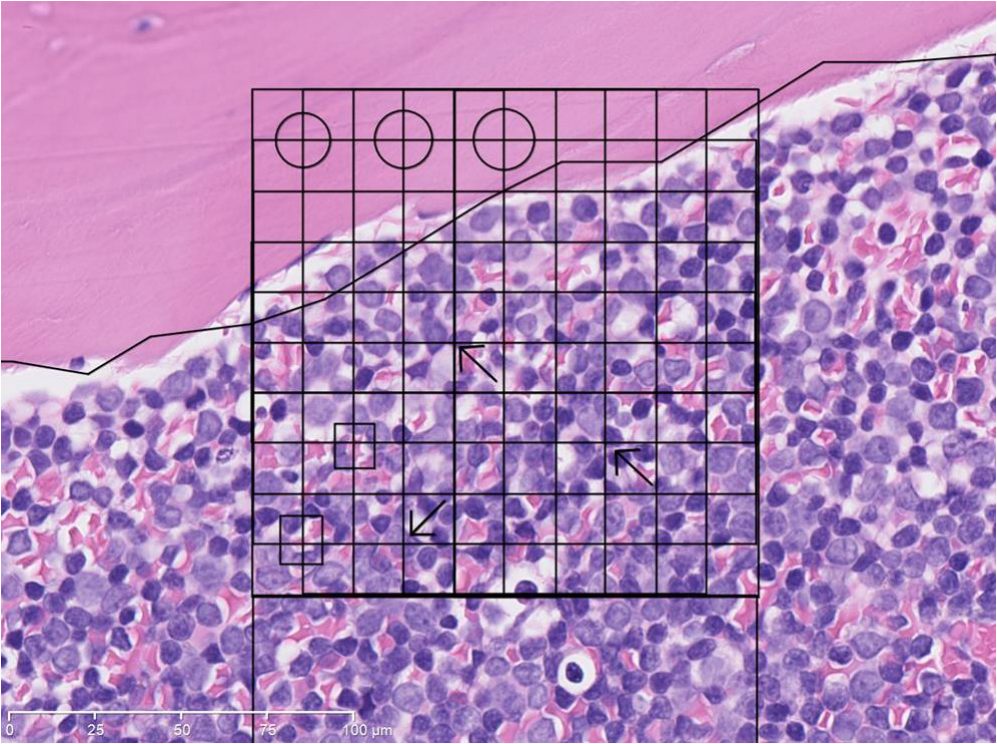
Trephine biopsy specimen from the diagnosis of ALL. Magnification x40. Circles mark trabecular bone, squares mark red cells, arrows mark leukemia cells.

Similarly, other bone marrow elements were counted when they lay under a crossing point of the grid. Bony trabeculae and leukemic/hemopoietic cells were easily identified at x40 magnification. Likewise, adipocytes, megakaryocytes and red cells were readily identifiable. Small vessels were identified based on the presence of endothelial cell lining. Sinusoids were only measured if there were cells present in their lumen. When the crossing point of a grid lay over none of the above identifiable component, it was called an ‘empty space’ (Fig 2).

Bone lining cells or osteoblasts adhered to the surface of bony trabeculae; osteoclasts were identified on the basis of multiple nuclei and close proximity to the trabecular surface. These two cell types were enumerated in 20 randomly chosen fields of 0.02 mm^2^ area along the surface of bony trabeculae.

In addition to quantifying bony trabecular elements under the grids, the trabeculae were also traced with an electronic pen and the surface area compared to that of the whole trephine biopsy specimen area. This measurement may give a more accurate measurement of the area occupied by bony trabeculae.

### Statistical analysis

The data are presented as means with 95% confidence intervals. The effects of Time (Baseline versus Day 15 versus Day 30) were evaluated using linear mixed effects models, with the outcome measure being repeated over time. Three outcomes—osteoblasts, fat cells and red cells—were log transformed prior to analysis due to violations of distributional assumptions of linear regression. The regression coefficients were back-transformed following analysis, with the result being that mean differences represent the ratio of two geometric means rather than as absolute differences between the means of the two groups. A fourth outcome—Osteoclasts—was treated as a count variable and was modeled using a mixed effects model with a Poisson distribution and a log link function. As with the log-transformed models, mean differences between groups represent the ratio of two means. Subject was treated as a random factor in all models. All effects were assessed at the p<0.05 alpha level of significance. The data were analyzed using SAS 9.3 (SAS Institute Inc., Cary, NC, USA).

## Results

Five of the patients with ALL presented with T-cell disease, 39 with precursor-B ALL. All patients attained remission. Thirty seven of the patients were evaluable for MRD; 35 had low MRD with <1x10^-3^ and two patients had high MRD with >1x10^-3^. Two of the 44 patients had a relapse during the observation period and one patient died of septicemia. The relatively low number of patients and the short follow-up period preclude further analysis of correlation between morphometric values and outcome.

### 1. Morphometric Evaluation of ALLs versus controls

The two patient groups were comparable in age distribution and gender (Table 1). Haemoglobin, neutrophil and platelet values were significantly lower while white blood cells significantly higher in patients with ALL, reflecting the extensive involvement of the marrow in ALL.

**Table 1:** Bone Marrow Biopsy results in patients with ALL and controls.

	ALL (Day 0)	Controls	Difference (p)
**Number of patients**	44	32	
**Sex (male: female)**	23:21	18:14	0.46
**Presentation features**			
**Age at diagnosis (years)**	6.3 (4.8–7.8)	7.4 (5.5–9.4)	0.33
**Hemoglobin (g/L)**	90.3 (84.6–96.0)	114.1 (108–120)	<0.0001
**Platelets (x10** ^**9**^ **/L)**	95.9 (68.9–122.9)	348.5 (305–392)	<0.0001
**WBC (x10** ^**9**^ **/L)**	27.5 (12.5–42.4)	10.4 (8.3–12.4)	0.03
**Absolute neutrophil count (x10** ^**9**^ **/L)**	1.7 (0.7–2.6)	6.0 (4.1–8.0)	<0.0001
**Absolute lymphocyte count (x10** ^**9**^ **/L)**	4.3 (3.2–5.3)	3.2 (2.5–3.8)	0.081
**Trephine biopsy specimen results**			
**Trephine biopsy surface area (mm** ^**2**^ **)**	10.0 (8.1–11.8)	10.0 (7.8–12.1)	0.998
**Bony trabecular surface area (% of whole biopsy specimen)**	22.9 (19.7–26.1)	28.3 (24.6–32.1)	0.03
**Trabecular width (μm)**	157 (136–178)	176 (151–200)	0.25
**Bony trabeculae** *	16.1 (13.8–18.4)	16.6 (13.9–19.3)	0.77
**Hemopoietic/leukemia cells** *	45.3 (40.6–50.0)	36.5 (31.1–42.0)	0.018
**Fat cells** * ^**#**^	0.7 (0.3–1.1)	10.9 (7.8–14.9)	<0.0001
**Empty spaces** *	7.3 (5.5–9.1)	8.9 (6.8–11.0)	0.24
**Small vessels** *	0.5 (0.3–0.7)	0.5 (0.2–0.7)	0.68
**Sinusoids** *	0.8 (0.1–1.5)	1.8 (1.0–2.6)	0.08
**Sinusoids width (μm)**	32.5 (25.9–39.1)	32.7 (25.3–40.2)	0.96
**Extracellular matrix** *	25.6 (22.7–28.5)	17.1 (13.7–20.5)	0.0003
**Red cells** * ^**#**^	1.6 (1.1–2.3)	2.3 (1.6–3.3)	0.18
**Megakaryocytes** **	2.7 (1.4–3.9)	11.3 (9.8–12.7)	<0.0001
**Reticulin fibres** *	17.7 (15.2–20.2)	5.7 (2.9–8.6)	<0.0001
**Osteoblasts** *** ^**#**^	15.7 (13.0–18.9)	55.6 (44.9–69.0)	<0.0001
**Osteoclasts** ****	1.80 (1.4–2.3)	3.1 (2.6–3.8)	0.0006

Mean values and 95% confidence intervals. Bony trabecular surface area is given as a percentage of trephine biopsy specimen surface area.

* Percentage of crossing points occupied by element.

** Megakaryocytes were counted inside 20 whole squares of 0.02 mm^2^ each.

*** Osteoblasts were counted in 10 whole squares of 0.02 mm^2^ each.

**** Counted along all trabecular perimeters of the whole trephine biopsy specimen area.

# Geometric means (see Statistical Methods).

P values have not been corrected for multiple observations.

The surface areas of trephine biopsies were similar in the two groups. The proportion of the trephine biopsy occupied by bony trabeculae as traced by an electronic pen was slightly lower in patients with ALL than controls. However, there was no difference between the two groups in the crossing points occupied by bony trabeculae or the width of these trabeculae as measured under the grids. Sinusoids were detected less frequently in some ALLs but the width of sinusoids was the same in both groups. There was more extracellular matrix in the leukemia samples (P<0.0003) and a significantly higher number of reticulin fibres (P<0.0001).

Cellularity, as expressed by the percentage of crossing points occupied by hemopoietic or leukemic cells was generally higher as predicted in the leukemia group, while adipocytes were significantly reduced compared to the control group (P<0.0001). There were substantial reductions in the numbers of both megakaryocytes (P<0.0001) and osteoblasts (trabecular bone lining cells (P<0.0001).

### 2. Morphometric indicators of bone marrow recovery

While there were no withdrawals from the study, acceptable trephine biopsies were obtained from fewer leukemia patients at day 15 or day 30 of therapy than at diagnosis, due to technical difficulties. (Table 2)

**Table 2:** Morphological Measures of Bone Marrow Recovery in ALL.

	At Dx	Day 15	Day 29/Day 33	Type III effect of time. P
**N** ^**o**^ **of samples**	44	26	30	
**Trephine biopsy specimen surface area (mm** ^**2**^ **)**	9.9 (8.4–11.5)	7.1 (5.1–9.1)	9.1 (7.2–10.9)	0.047
**Bony trabecular surface area as percentage of trephine biopsy surface**	22.9 (19.6–26.1)	25.6 (21.4–29.9)	25.8 (21.8–29.8)	0.40
**Trabecular width (μm)**	157 (140–175)	166 (143–188)	149 (127–171)	0.56
**Bony trabeculae** *	16.1 (13.6–18.6)	17.6 (14.3–21.0)	19.8 (16.8–22.8)	0.18
**Hemopoietic/leukemia cells** *	45.3 (39.6–50.9)	21.5 (17.0–26.0)	14.5 (11.6–17.5)	<0.0001
**Fat cells** * ^**#**^	0.6 (0.3–1.1)	2.4 (1.5–3.8)	16.2 (11.7–22.3)	<0.0001
**Empty spaces** *	7.3 (5.6–8.9)	13.9 (10.1–17.8)	13.4 (10.3–16.5)	0.0002
**Small vessels** *	0.5 (0.3–0.7)	0.3 (0.04–0.5)	0.4 (0.1–0.6)	0.26
**Sinusoids** *	0.8 (0.2–1.4)	1.7 (0.9–2.5)	1.4 (0.8–2.1)	0.15
**Sinusoid width (μm)**	32.5 (27.3–37.6)	49.4 (43.2–55.7)	46.7 (40.7–52.8)	0.0001
**Extracellular matrix** *	25.7 (22.3–29.0)	34.7 (30.4–38.9)	23.7 (19.7–27.7)	<0.0001
**Red Cells** * ^**#**^	1.6 (1.1–2.3)	4.0 (2.6–5.9)	3.2 (2.1–4.6)	0.002
Megakaryocytes**	2.7 (1.4–4.0)	5.5 (4.0–7.1)	3.2 (1.72–4.73)	0.013
**Reticulin fibres ** *	17.6 (15.2–20.0)	12.3 (9.0–15.6)	7.9 (5.0–10.8)	<0.0001
**Osteoblasts** *** ^**#**^	15.5 (12.7–19.0)	14.6 (10.7–19.9)	14.8 (12.5–17.6)	0.89
Osteoclasts****	1.6 (1.1–2.2)	1.0 (0.7–1.6)	1.2 (0.8–1.9)	0.27

Mean values and 95% confidence intervals given.

* Percentage of crossing points occupied by element.

** Megakaryocytes were counted inside 20 whole squares of 0.02 mm^2^ each.

*** Osteoblasts were counted in 10 whole squares of 0.02 mm^2^ each.

**** Counted along all trabecular perimeters of the whole trephine biopsy specimen area.

# Geometric means (see Statistical Methods).

**Fig 3:**
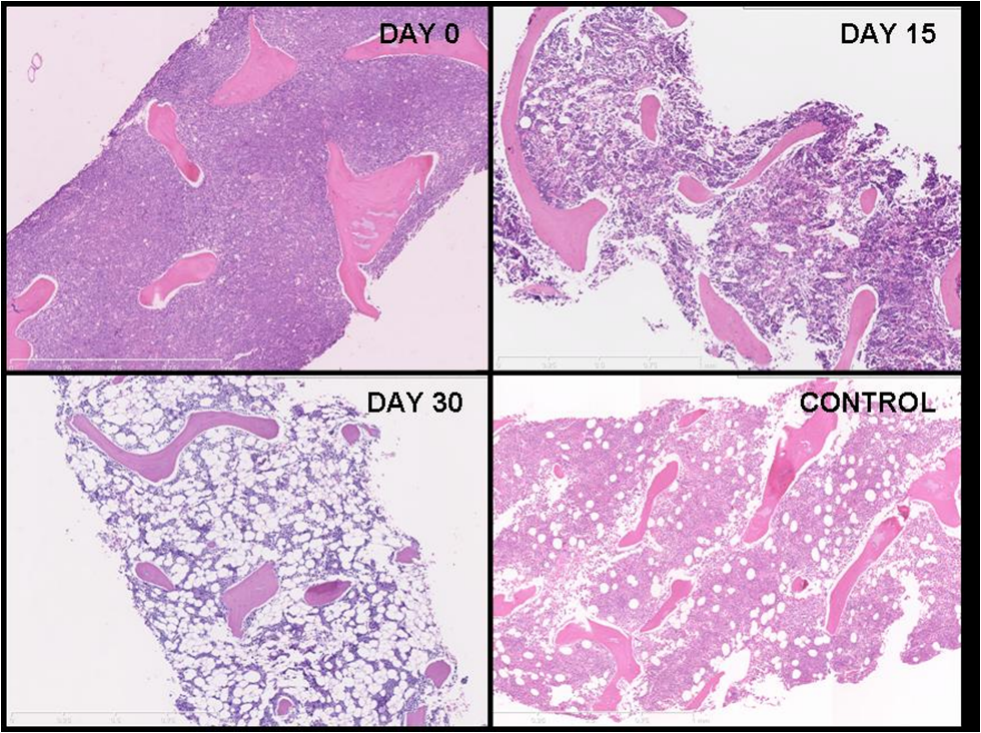
Morphologic changes in the marrow of a patient with ALL and a control patient. Marrow recovery for patient: ALL UPN 21/2010 (Days 0, 15 and 30) and for Control patient: UPN 72/2011.

There were several differences observed in the marrow from diagnosis through day 15 to the end of induction treatment. The cellularity progressively decreased, reflecting the clearance of leukemia cells from the marrow particularly in the first two weeks (d0 to d15 P<0.0001). In parallel, the adipocyte count increased significantly (d0 to d15 P = 0.0001) (Fig 3). Some changes in the sinusoids were observed with an increase in sinusoid width by day 15 (P = 0.0002). There was a rise in the amount of extracellular matrix or amorphous tissue from day 0 to day 15 (P = 0.0004), probably reflecting an increase in necrotic material due to chemotherapy induced cell death and /or serum proteins occupying the spaces created. This transient rise was followed by a significant reduction from d15 to d30 (P<0.0001). Red cell numbers increased by day 15, although this could reflect the fragility of the trephine biopsy sites (d0 to d15 P = 0.001) and megakaryocyte numbers showed a transient improvement (d0 to d15 P = 0.005). Reticulin fibrosis was much less evident at day 15 (P<0.005) with a further reduction at day 29/33. In contrast, osteoblast and osteoclast numbers were unchanged during remission induction.

## Discussion

At the diagnosis of ALL, the bone marrow is characterized by heavy infiltration by leukemic blast cells that occupy most of the marrow. This is accompanied by increased reticulin fibrosis that may serve to ‘anchor’ the leukemic cells in the marrow [2,3]. Moreover, the area occupied by bony trabeculae was reduced in ALL trephine biopsy specimens compared to those from patients with other cancers. Leeuw et al found that bone volume and trabecular thickness were reduced in ALL patients less than 10 years of age at diagnosis but there was no difference in older patients [7]. Our data showed a significant difference in the bony trabecular surface area between ALL patients and controls but there was no difference between patients less or more than 10 years at diagnosis.

In keeping with the studies of Noren-Nystrom [1], we found significantly increased bone marrow fibrosis in diagnosis bone marrow biopsies of patients with ALL. The increased marrow stiffness could play a role in leukemia progression [8] and could potentially be targeted with compounds which modify the expression and activity of enzymes such as lysyl oxidase, which regulate extracellular matrix (ECM) remodeling and degradation [9].

The area in the marrow occupied by adipocytes was greatly reduced at diagnosis as compared to controls. From the current study, it is unclear whether this decrease in adipocyte volume relates to a decrease in adipocyte number or a reduction in the degree of lipid accumulation within each adipocyte. Bone marrow adipocytes have been implicated in the early phases of proliferation in multiple myeloma possibly through leptin secretion and may play a role in the growth of ALL blasts. Adipocytes then tend to disappear during disease development [10].

Patients who present with severe bone pain tend to have more normal blood counts than those who present without [11], [12]. In our analyses, bone pain as a presentation symptom prior to diagnosis was associated with a lower WBC and peripheral blood blast cell count but no difference in the percentage of marrow occupied by bony trabeculae.

The other significant differences in morphology between ALL patients and controls (cellularity, fat cells, extracellular matrix (ECM) and the number of megakaryocytes) are reflections of the bone marrow infiltration and the absence of normal haemopoiesis. ECM components are essential for normal hematopoietic developmental processes and regulates a wide range of cellular functions in both normal and in malignant tissue as reviewed by Lu et al [13]. Future studies examining the composition and development potential of cells comprising the bone marrow stroma could shed light on the current findings regarding the ALL associated bone marrow changes.

Importantly, we found a significant difference in the number of osteoblasts that play an important role in providing support for hemopoietic stem cells (HSC) and also the number of osteoclasts [14] [15]. Our current studies are looking at the identification of cytokines that may play a role in this stromal suppression. Recent data suggest a reciprocal relationship between osteoblasts in the marrow and leukemia progression [16]. The leukemic process leads to a diminution of osteoblasts. Reinstatement of the osteoblast numbers and function by pharmacological means can reduce leukemia burden. Osteoclasts in our analysis were also depressed in numbers from which we conclude that the leukaemic process effects both these cell types more or less equally which would explain why we do not usually see much osteoporosis at diagnosis.

The other major aim of the morphometry study was to analyze the changes in the marrow of ALL patients that take place during the first month of treatment. We were in a unique position to study these changes since serial trephine biopsies were done routinely in our department. Our findings showed some major changes occurring during remission induction. In addition to the rapid disappearance of leukemic blast cells, there was a significant reduction in reticulin fibrosis. This latter observation confirms the findings of Noren-Nystrom et al [1]. The reduction in leukemia cells was associated with a significant increase in the areas occupied by adipocytes. Similarly, there was a significant increase in the width of sinusoids which are also thought to play an important role in the maturation and egress of HSCs from the bone marrow [17].

The functional integrity of the supporting scaffolds (cancellous bone and adipocytes) and vascular structures seems to be essential for normal hematopoiesis [18]. A reduced cancellous bone surface and sinusoid surface may negatively influence normal osteogenic differentiation of mesenchymal stem cells, impair hematopoiesis and result in undifferentiated fibroblasts and increased levels of reticulin fibres. Potential new agents targeting bone marrow stroma could confer sensitivity to chemotherapy in resistant cell populations [19]. While intensified chemotherapy has lead to a dramatic increase in ALL cure rates, it is reaching its upper limits of efficacy, with further intensification likely to lead to increased toxicity. Identifying potential new targets in the bone marrow stroma holds the promise of attacking the leukemic process from another angle.

A note of caution regarding the use of morphometry should be added: the use of the described grids for the quantitation of marrow elements is very time consuming and still remains less reliable for some components than oriented counting. Tracing cancellous bone e.g. or measuring the width of sinusoids was a more reliable approach than using the percentage of that component under the grids. Bone lining osteoblasts could only be reliably quantitated using an oriented approach. Perhaps a fully automated, computerized method, such as laser scanning cytometry may reveal more robust changes, especially if combined with immunohistochemistry [20].

## Conclusions

The pattern that emerges from our morphometric studies of the bone marrow recovery process is consistent with the notion that the disappearance of leukemic cells drives the dynamic changes that take place in the marrow cavity during the first four weeks of chemotherapy. Similarly to the decrease in leukemic cell infiltrates, reticulin fibrosis returned to almost normal levels by the end of induction. While it remains to be determined what drives these changes, either a reduction in leukemia cell-derived cytokines, chemokines and adhesion molecules or changes in the availability of nutrients and oxygen tension may be important in facilitating the normal hemopoietic expansion processes required for recovery [21].

## Supporting Information

S1 Dataset De-identified clinical and morphometry data. This file contains all the important clinical, laboratory and morphometry data.
(XLSX)
